# Supporting data for the effect of gamma-secretase inhibitors in osteoclast differentiation and spreading

**DOI:** 10.1016/j.dib.2016.03.018

**Published:** 2016-03-10

**Authors:** Won Jong Jin, Bongjun Kim, Jung-Wook Kim, Hong-Hee Kim, Hyunil Ha, Zang Hee Lee

**Affiliations:** a Department of Cell and Developmental Biology, Dental Research Institute, School of Dentistry, Seoul National University, Seoul 110-749, Republic of Korea; b Clinical Research Division, Korea Institute of Oriental Medicine, Daejeon 305-811, Republic of Korea

**Keywords:** Osteoclast, Notch, GSI, Differentiation, Spreading

## Abstract

The data in this article is related to the research article entitled “Notch2 signaling promotes osteoclast resorption via activation of PYK2” (Jin et al., 2016 [1]). To block Notch signaling activation, we used several gamma-secretase inhibitors (GSIs) and evaluate the inhibitory potential of GSIs on osteoclastogenesis. We measured the effect of GSIs on osteoclastogenesis and normal spreading of osteoclasts by using the mouse bone marrow-derived macrophages (BMMs) which may contributes to insight of physiological relevant of in vivo. This data article suggests valuable approach to GSIs treatment doses and potential of those in the osteoclast differentiation and spreading.

**Specifications Table**

**Table:** 

Subject area	Biology
More specific subject area	Developmental biology
Type of data	Figure, Graph
How data was acquired	Cell culture, TRAP-stain, Microscope, OsteoMeasure XP program (version 1.01; OsteoMetrics).
Data format	Analyzed
Experimental factors	Bone marrow cells were obtained by flushing tibiae of mice and nonadherent cells were expanded for three days using M-CSF to generate Bone marrow-derived macrophage, a progenitor of osteoclast.
Experimental features	Bone marrow-derived macrophages were differentiated osteoclast with various dose and several gamma-secretase inhibitors in the presence of M-CSF and RANKL. Fully differentiated osteoclasts were TRAP-stained to measure osteoclast number and spreading.
Data source location	Seoul National University, Seoul, Republic of Korea
Data accessibility	Data with this article

## Value of the data

• This data shows the effect of gamma-secretase inhibitors (GSIs) on osteoclast formation and normal spreading.
• Various doses of GSIs provide comparison of inhibitory potential on osteoclast differentiation and spreading.
• This data provides starting and target doses for several GSIs against osteoclast differentiation and spreading that offers valuable approach for investigation of Notch signaling and gamma-secretase involvement in osteoclast differentiation and cytoskeletal organization.

## 1 Data

This data provides supporting information of the role of Notch signaling on osteoclast differentiation and function [1]. Notch signaling has been shown to regulate osteoclastogenesis negatively by Notch1 or positively by Notch2 [2]. To investigate whether Notch signaling affects osteoclast differentiation and spreading, we assessed the inhibitory potential of four GSIs from BMM to mature osteoclast forming period.

## 2 Experimental design, materials and methods

### 2.1 Reagents

Recombinant human M-CSF and RANKL were purchased from PeproTech EC (London, UK). The gamma-secretase inhibitors, Dibenzazepin (PubChem CID: 11454028), L685,458 (PubChem CID: 5479543), Compound E (PubChem CID: 11306390) DAPT (PubChem CID:5311272) were purchased from Calbiochem-Merck Co (Darmstadt, Germany).

### 2.2 Cell culture

To obtain BMMs, bone marrow cells (BMCs) were collected by flushing tibiae from 5-week-old male ICR mice and red blood cells had been removed with ACK buffer (0.01 mM EDTA, 0.011 M KHCO_3_, and 0.155 M NH_4_CL, pH 7.3). Cells that had not attached to the culture dish were further cultured for three days in the presence of M-CSF (60 ng/ml) alone to generate BMM as previously described [3].

To generate osteoclast, BMMs (4×10^4^ cells/well) were cultured in α-minimum essential medium (α-MEM) containing 10% (v/v) heat inactivated fetal bovine serum (FBS), 50 U/ml penicillin, and 50 μg/ml streptomycin on 48-well culture plates in the presence of M-CSF (60 ng/ml) and RANKL (100 ng/ml) for 4 days. The complete medium was changed every 3 days.

### 2.3 TRAP stain and measurement

**Fig. 1:**
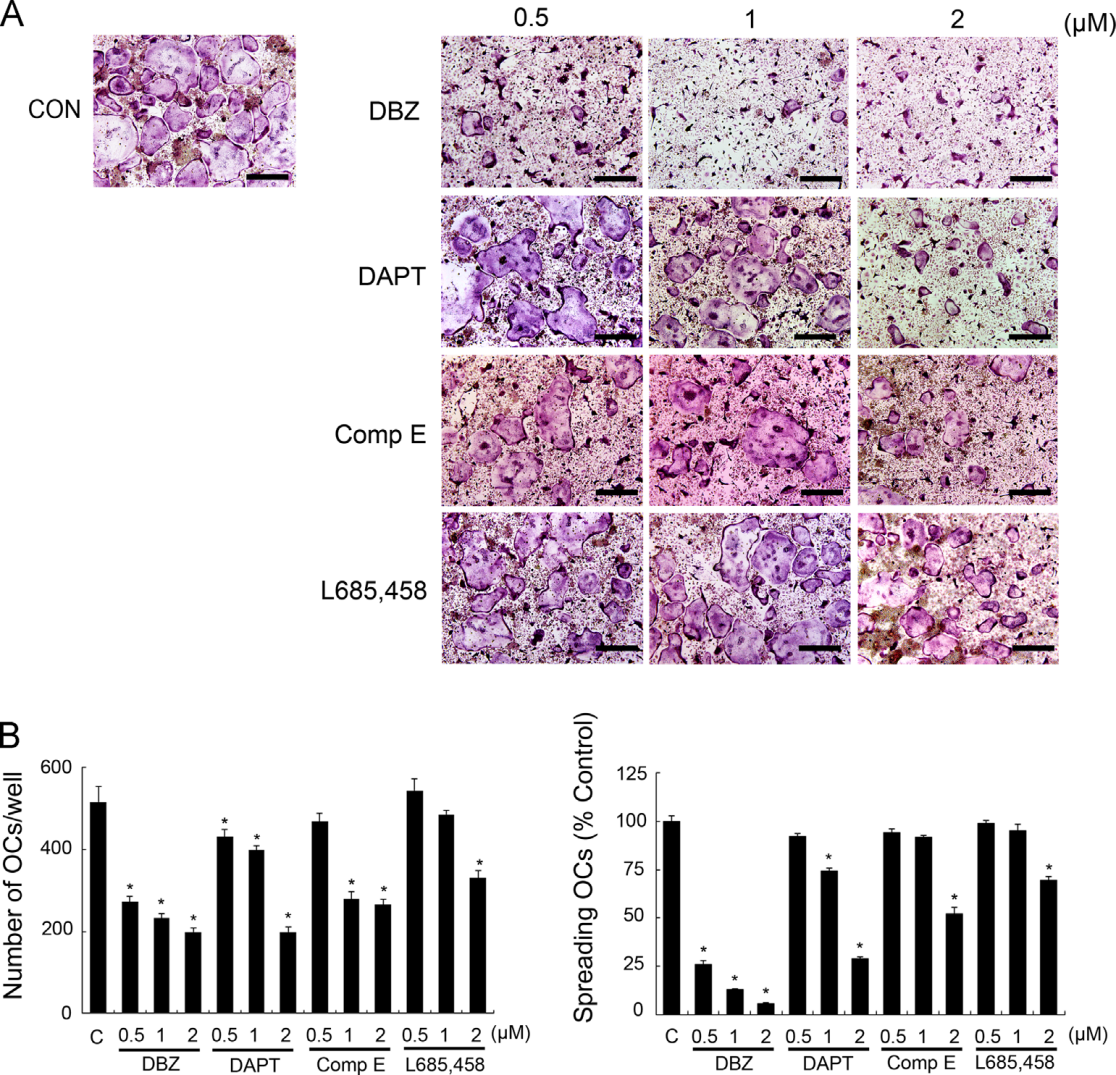
GSIs inhibit osteoclast differentiation and spreading. (A–C) BMMs were cultured with DMSO or the indicated dose of several GSIs in the presence of M-CSF (60 ng/ml) and RANKL (100 ng/ml) for 4 days. (A) After culturing, the cells were stained for TRAP. Scale bar is 100 μm. (B) Osteoclasts that contained three or more nuclei were counted (left) and the number of spreading osteoclasts was measured (right) (^⁎^*P*<0.05).

Appeared osteoclasts were washed by PBS and fixed by 3.7% formalin for 20 min. Then, fixed cells were stained for tartrate-resistant acid phosphatase (TRAP) by using the Leukocyte Acid Phosphatase Assay kit (Sigma-Aldrich) following the manufacturer׳s procedure (Fig. 1A). DMSO- or several GSIs-treated osteoclasts that contained three or more nuclei were counted by using of light microscope and the number of spreading osteoclasts was measured by using the OsteoMeasure XP program (version 1.01; OsteoMetrics) (Fig. 1B).

### 2.4 Statistical analysis

All quantitative data are represented as means±SDs (*n*≥3). Each experiment was performed 3–5 times, and the results from 1 representative experiment are shown. Statistical differences were analysed by Student׳s *t-*tests. A value of *P*<0.05 was considered statistically significant.

## References

[1] Jin W.J., Kim B., Kim J.W., Kim H.H., Ha H., Lee Z.H. (2016). Notch2 signaling promotes osteoclast resorption via activation of PYK2. Cell Signal..

[2] Duan L., Ren Y. (2013). Role of notch signaling in osteoimmunology—from the standpoint of osteoclast differentiation. Eur. J. Orthod..

[3] Kim S.D., Kim H.N., Lee J.H., Jin W.J., Hwang S.J., Kim H.H. (2013). Trapidil, a platelet-derived growth factor antagonist, inhibits osteoclastogenesis by down-regulating NFATc1 and suppresses bone loss in mice. Biochem. Pharmacol..

