# Supplementary material for: Supporting data for the effect of gamma-secretase inhibitors in osteoclast differentiation and spreading
Source: Data Brief. 2016 Mar 10;7:682–5. doi: 10.1016/j.dib.2016.03.018 (PMC4802519; doi:10.1016/j.dib.2016.03.018)
Supplement: Supplementary file 1 — Supplementary material [file mmc1.doc]

**Conflict of interest**

None.
